# A new clustered federated learning algorithm for heterogeneous data in high-precision wireless sensing

**DOI:** 10.3389/frai.2026.1718193

**Published:** 2026-02-04

**Authors:** Zongrui Tian, Jiasheng Tian

**Affiliations:** School of Electronic Information and Communications, Huazhong University of Science and Technology, Wuhan, China

**Keywords:** data heterogeneity, federated learning algorithm, KL divergence, personalized federated learning algorithm, wireless sensing

## Abstract

**Introduction:**

This article studies a new clustering-based federated learning algorithm that leverages Kullback-Leibler (KL) divergence to tackle heterogeneous data in wireless sensing environments.

**Methods:**

Firstly, highdimensional heterogeneous data is subjected to principal component analysis to generate dimension-reduced representations, thereby reducing computational complexity. Secondly, the KL divergence distances between each pair of clients are calculated, followed by clustering according to the minimum threshold. The new KL divergence distance between the aggregated clients and others is taken as the average of the two. Finally, the federated learning training is conducted within each cluster to obtain a personalized model based on the classic wireless datasets.

**Results and Discussion:**

After the personalized models are tested, clients are reclustered and the models are updated—that is, a series of iterative operations, the optimal number of clusters and recognition accuracy are obtained. The test results show that the proposed algorithm based on KL divergence has higher recognition accuracy than several reported ones.

## 1 Introduction

With the rapid development of Internet of Things (IoT) technology and 6G network architecture, wireless sensing technology has been widely applied in scenarios such as smart home, industrial monitoring, health care and environmental perception. These applications generate massive amounts of sensing data that contain valuable information for decision-making. However, these raw data scattered across various devices are directly transmitted to cloud computing centers for centralized processing, resulting in high system communication overhead and a potential threat in safeguarding user data privacy.

However, traditional federated learning (FL) algorithms (e.g., FedAvg) assume that data across clients follows an Independent and Identically Distributed (IID) distribution, which is difficult to satisfy in practical wireless sensing environments. Due to differences in sensing device types, deployment locations, and working conditions, data among clients often exhibits significant heterogeneity (i.e., Non-IID), including distribution shifts, feature space discrepancies, and label distribution imbalance. This heterogeneity leads to performance degradation of the globally shared model.

To tackle the Non-IID problem in FL, personalized federated learning (PFL) has emerged as a research hotspot. Existing personalized methods can be roughly divided into three categories: (1) regularization-based methods, which introduce additional regularization terms to constrain the local model updates, balancing the consistency between local and global models; (2) model adaptation-based methods, which adjust specific components of the global model or fine-tune local model parameters to adapt to local data; (3) clustering-based methods, which group clients with similar data characteristics into clusters, and train cluster-specific models to achieve personalization. Among these, clustering-based methods are particularly suitable for wireless sensing scenarios due to their low computational overhead and strong scalability. However, existing clustering strategies in FL mainly rely on similarity metrics such as cosine similarity or Euclidean distance, which focus on feature space distance but fail to effectively capture the distribution differences between heterogeneous sensing data. This limitation leads to inaccurate clustering results, thereby affecting the performance of personalized models.

Kullback–Leibler (KL) divergence is a classic metric for measuring the difference between two probability distributions, which can quantitatively characterize the distribution deviation of heterogeneous data. Compared with cosine similarity (which focuses on direction consistency) and Euclidean distance (which focuses on feature value difference), KL divergence is more suitable for describing the intrinsic heterogeneity of sensing data. Motivated by this, this article proposes a KL divergence-based PFL method for wireless sensing environments.

The main contributions of this article are summarized as follows: (1) a clustering strategy based on KL divergence is proposed to effectively capture the distribution differences of heterogeneous sensing data, improving the accuracy of client clustering compared with traditional similarity metrics; (2) a PFL framework for wireless sensing is designed, which realizes model customization through cluster-specific training and iterative optimization, adapting to the data heterogeneity of sensing human behaviors; (3) extensive experiments are conducted on two classic wireless sensing datasets to verify the effectiveness of the proposed PFL algorithm based on KL divergence. The results demonstrate that the algorithm outperforms serval reported algorithms in terms of recognition accuracy.

## 2 Related work

Federated learning (FL) offers a solution to the challenge of scattered data hindering the centralized learning. In McMahan et al. (2017), first proposed the concepts of FL and verified its effectiveness and feasibility for collaborative model training without aggregating user data to a central server (Sattler et al., 2019). However, in data-heterogeneous environments, FL algorithms often suffer from significant performance degradation (Zhao et al., 2018). To address the challenge of data heterogeneity (Pang et al., 2025a,b), many researchers have proposed a range of improved methods such as personalized federated learning (PFL) algorithms (Tan et al., 2022; Arivazhagan et al., 2019).

One personalized approach involves designating some layers of a neural network as personalized layers and the rest as globally shared layers (Arivazhagan et al., 2019; Liang et al., 2022). In Arivazhagan et al. (2019), proposed the FedPer algorithm, which adopted a “base layers + personalized layers” design. In Liang et al. (2022), proposed LG-FedAvg, where the last several layers of the neural network were designated as personalized components. However, how to properly divide base layers and personalized layers in these algorithms remains an area requiring further research. In Collins et al. (2021), proposed the FedRep algorithm and designed the classification head of a neural network as the personalized component, while all other layers were designed for global federated training. Nevertheless, FedRep’s performance depends on the effectiveness of global representations; moreover, in real-world scenarios, if shared features across data from different clients are either less prominent or difficult to learn, this algorithm is at the risk of performance degradation.

Another personalized approach is that each personalized model exhibits a certain “degree of personalization” relative to the global model (Dinh et al., 2020; Deng et al., 2003; Li et al., 2021; Zhang et al., 2020). In Dinh et al. (2020), proposed the PFL algorithm “pFedMe” and introduced a regularized loss term to balance the trade off between personalization and generalization. However, pFedMe faced challenges in properly selecting hyperparameters to quantify this degree of personalization. In the same year, Deng et al. (2003) introduced an adaptive weight adjustment mechanism to dynamically tune the weight ratio between the global model and local personalized model in the final model. However, the adaptive weights are dynamically determined based on the loss, leading to insignificant improvement effects. In Li et al. (2021), proposed the Ditto algorithm, which employed the traditional FedAvg method for global model optimization. During synchronous training, FedAvg adopted a relatively global regularized model as the local personalized model. Nevertheless, using FedAvg for global training is unfavorable for convergence in data-heterogeneous scenarios, and Ditto also faces challenges in selecting hyperparameters to quantify the degree of personalization.

The aforementioned PFL algorithms generally focus on the personalized components of individual models relative to the global model, but do not directly consider the connections between two personalized models-specifically, the similarity among models across multiple clients (Zhang et al., 2020; Huang et al., 2021).

In Zhang et al. (2020), proposed the FedFomo algorithm, which achieved personalized updates by computing the optimal weighted combination of models for each client. Each client determined its aggregation weights based on the local loss of other clients’ models, resulting in models with lower losses being assigned larger weights. This algorithm improves model performance only to a certain extent. In Huang et al. (2021), put forward the FedAMP algorithm, which emphasized attention mechanisms to enhance pairwise collaboration among clients with similar data distributions. However, these personalized models accounting for the connections between personalized models tend to exhibit notable similarity as a result of such collaborative interactions.

Additionally, numerous studies Briggs et al. (2020), Islam et al. (2024), Ghosh et al. (2020), and Sattler et al. (2020) have focused on clustering based on personalization in FL. For this category of algorithms, the server initially randomly constructed K global models based on a certain type of similarity (e.g., distance similarity, cosine similarity), each associated with a distinct cluster. However, the clustering algorithm (Ghosh et al., 2020) required predefining the number of clusters K and involved frequent communication for transmitting model parameters or frequently varying them. Secondly, many relevant hyperparameters such as thresholds and cluster partitioning conditions were involved, leading to a linear increase in complexity (Sattler et al., 2020).

Therefore, this article studies a new clustered FL algorithm to address the challenge of data heterogeneity—specifically by leveraging Kullback–Leibler (KL) divergence to measure the similarity among multiple clients and performing effective clustering via iterative loops. This new algorithm enables efficient identification of distribution similarity, avoids the server’s arbitrary initial determination of K global models, and both difficulties in hyperparameter selection and the need for frequent communication.

## 3 Materials and methods

This section will introduce the new clustered federated learning (CFL) algorithm and datasets.

### 3.1 The new CFL algorithm

The New CFL algorithm includes the Principal Component Analysis (PCA), KL divergence calculation, clustering and federated learning training, etc.

#### 3.1.1 Principal Component Analysis

Principal Component Analysis (PCA) is a statistical method that projects data onto a low-dimensional space via linear transformation, while maximizing the retention of the variance of the original data. In the new CFL algorithm, each client derives a principal component vector matrix using PCA based on its local data; this matrix is regarded as the client’s data feature and is used to measure the distance between different clients. Specifically, for each client’s data matrix *X* ∈ *R*^*M* × *d*^, after standardization and subsequent PCA processing (including covariance calculation, eigenvalue computation, and eigenvector derivation), a principal component vector matrix *U* ∈ *R*^*d* × *c*^ is obtained. Here, *M* denotes the number of samples, *d* represents the feature dimension of a single sample, and *c* is the selected number of principal components (with *c* < *d*). *c* is a user-specified value, which is used to determine the dimension after dimensionality reduction. This process can be expressed as:


U=PCA(X,c) (1)

After finishing PCA, we obtain the data features for each client. These data features are typically in the form of matrices, so the matrix vectorization for these data is required to simplify the subsequent KL divergence calculations. Specifically, we vectorize the matrix *U* ∈ *R*^*d* × *c*^ into *U* ∈ *R*^1 × (*d* × *c*)^ (either a column vector or a row vector). Following the matrix vectorization, each client is assigned a corresponding characteristic vector *U*. We then take the absolute value of *U* and normalized it, as can be given by


$$U_{i}=\frac{\left|U_{ij}\right|}{\overset{d\times c}{\underset{j=1}{\sum }}\left|U_{ij}\right|}$$ (2)

Where *i* = 1, 2, …, *N* represents the number of the client numbers. And thus *U* is converted into the discrete probability distribution *U*.

Since the heterogeneous wireless sensing data *X* including high dimensionality, excessive redundant information, and noise interference, it is necessary for PCA, a classical dimensionality reduction and data preprocessing technique, to map high-dimensional variables to a low-dimensional principal component space via orthogonal transformation while preserving key variation information within the dataset. The low-dimensional data after PCA dimensionality reduction can not only reduce the computational cost of KL divergence and improve clustering efficiency, but also enhance the recognition sensitivity of KL divergence to “category differences in heterogeneous data.”

#### 3.1.2 Calculation of KL divergence

Kullback–Leibler (KL) Divergence is a method for measuring the difference between two probability distributions. The value of KL divergence is non-negative: it equals zero if the two probability distributions are identical, and the smaller the value, the more similar the distributions. The preprocessed vectors *U* are treated as discrete probability density distribution, and thus two client’s KL divergence is calculated by


$$\mathrm{KL}\left(U_{m}\left(x\right),U_{n}\left(y\right)\right)=\overset{c}{\underset{i=1}{\sum }}x_{i}\log\left(\frac{x_{i}}{y_{i}}\right)$$ (3)

where *U_m_*(*x*) ∈ *R^c^* and *U_n_*(*y*) ∈ *R^c^* are two (*m*, *n*) client’s PCA vectors, and *x_i_*, *y_i_* denote the *i*-th component of the *m*-th client vector *U_m_*(*x*) and the *n*-th client vector *U_n_*(*y*), respectively.

#### 3.1.3 Clustering based on KL divergence

The hierarchical clustering algorithm is a clustering method that constructs a hierarchical structure of clusters through the gradual merging of existing clusters. Specifically, it determines the clustering relationships among clients based on the inter-client distance adjacency matrix *B*, as given by


$$B=\left[\begin{array}{cccc} {\mathrm{KL}}_{11} & {\mathrm{KL}}_{12} & \ldots & {\mathrm{KL}}_{1n}\\ {\mathrm{KL}}_{21} & {\mathrm{KL}}_{22} & \ldots & {\mathrm{KL}}_{2n}\\ \ldots & \ldots & \ldots & \ldots \\ {\mathrm{KL}}_{m1} & {\mathrm{KL}}_{m2} & \ldots & {\mathrm{KL}}_{mn} \end{array}\right]$$ (4)

where *m* = *n* generally is the number of clients. First, each client is treated as an individual cluster. Next, using the similarity (or distance) adjacency matrix, locate the two clusters with the highest similarity and merge them into a new cluster. Then, update the similarity adjacency matrix to accurately reflect the structure of the newly formed cluster. Finally, repeat the processes of cluster merging and similarity adjacency matrix updating until either the preset number of clusters or hyperparameter *cr* (clustered ratio defined as the ratio of the number of clustering operations to the total number of clients *N*) is attained or all values in the adjacency matrix are above the designated threshold. If KL_12_ is the minimum value among all KL*_mn_*, client 1 and client 2 are merged into a new client 1, and the number of clients is reduced to *m* − 1(=*n* − 1). The new KL divergence distance KL_1*j*_ between the new clustering client 1 and other clients *j* will be replaced by the average value of the KL divergence distance between the original two client (1, 2) and other clients *j*(*i*). Equation 4 can be updated to


$$B^{‘}=\left[\begin{array}{cccc} {\mathrm{KL}}_{11}^{\prime } & {\mathrm{KL}}_{12}^{\prime } & \ldots & {\mathrm{KL}}_{1\left(n-1\right)}^{\prime }\\ {\mathrm{KL}}_{21}^{\prime } & {\mathrm{KL}}_{22}^{\prime } & \ldots & {\mathrm{KL}}_{2\left(n-1\right)}^{\prime }\\ \ldots & \ldots & \ldots & \ldots \\ {\mathrm{KL}}_{\left(m-1\right)1}^{\prime } & {\mathrm{KL}}_{\left(m-1\right)2}^{\prime } & \ldots & {\mathrm{KL}}_{\left(m-1\right)\left(n-1\right)}^{\prime } \end{array}\right]$$ (5)

where


$${\mathrm{KL}}_{11}^{\prime }=0$$ (6)

$${\mathrm{KL}}_{1j}^{\prime }=\left({\mathrm{KL}}_{1\left(j+1\right)}+{\mathrm{KL}}_{2\left(j+1\right)}\right)/2,\;\;j=2,\ldots ,n-1$$ (7)

$${\mathrm{KL}}_{i1}^{\prime }=\left({\mathrm{KL}}_{\left(i+1\right)1}+{\mathrm{KL}}_{\left(i+1\right)2}\right)/2,\begin{array}{cc} & i=2,\ldots ,m-1 \end{array}$$ (8)

and


$${\mathrm{KL}}_{ij}^{\prime }={\mathrm{KL}}_{\left(i+1\right)\left(j+1\right)}\begin{array}{cc} , & i=2,\ldots ,m-1;j=2,\ldots ,n-1 \end{array}$$ (9)

If ${\mathrm{KL}}_{ij}^{\prime }$ (*i* = 1, 2, …, *m*−1; *j* = 1, 2, …, *n*−1) is still less than the minimum threshold *T*, the operation of clustering from Equation 4 to Equation 5 will be carried out once again. This course of clustering can be expressed by.


$$C=\left\{{\mathrm{CLU}}_{i}|i=1,2,3,\ldots \right\}=\mathrm{cluster}\left(B,T,\max\mathrm{num}\right)$$ (10)

which is the clustering set. And *T* is the threshold, maxnum is the maximum number of clustering operation. CLU*_i_* is also a set that contains the id of the clients in this cluster. It can be given as


$${\mathrm{CLU}}_{i}=\left\{{\mathrm{cid}}_{i,j}|j=1,2,3,\ldots \right\}$$ (11)

#### 3.1.4 Federated learning training based on *KL*

The traditional federated learning algorithm FedAvg is executed within each cluster and the personalized federated learning algorithm is carried out among different clusters, meaning there is no longer any interaction of model parameters between different clusters, with only clients within the same cluster interacting with each other’s model parameters.

For client *i*, the model (*w*_i_) is updated locally, the updating formula is expressed by


$$w_{i}^{t+1}=\underset{w_{i}^{t}}{\arg\min}\left[f_{i}\left(w_{i}^{t}\right)\right]$$ (12)

where *f*_i_ (*) is loss function. When the gradient descent method is adopted, the iteration model


$$w_{i}^{t,e+1}=w_{i}^{t,e}-\eta \frac{\partial f_{i}\left(w_{i}^{t,e}\right)}{\partial w_{i}^{t,e}}$$ (13)

will be executed repeatedly. And *η* is learning rate, *e* stands for epoch *E*. The server performs a weighted average (*p_i_*)based on the updated models of each client within the same cluster (*cluid*). The updated formula $w_{cluid}^{t+1}$ is as follows:


$$w_{cluid}^{t+1}=\underset{i}{\sum }p_{i}w_{i}^{t+1}$$ (14)

where *t* is the number of updating.

The server sends the global model $w_{cluid}^{t+1}$ to the clients; each client updates the model parameters and trains the model using local data; then send their local models to the server, which aggregates them. The algorithm can be carried out as the following steps: 
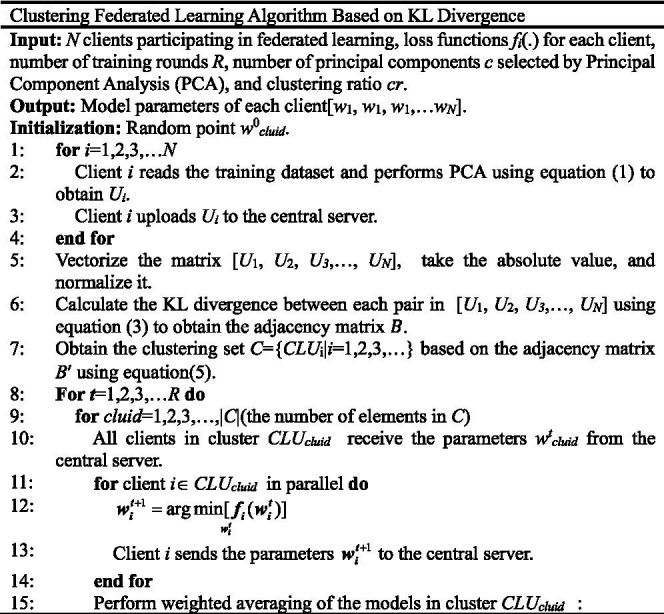


According to Equations 1–14, we can extract stable feature representations from WiFi CSI (Channel State Information) data or wireless dataset and obtain low-dimensional feature vectors by applying PCA/KL transform. And then we calculate the Kullback–Leibler (KL) divergence of CSI features between clients to generate a similarity matrix. Finally, we obtain a federated learning architecture for collaborative training among clients within each cluster. Each cluster trains a dedicated model to adapt to the signal propagation characteristics of specific areas. The model or local models tailored to a specific sub-distribution of clients could be used to better handle target-oriented sensing tasks where data distribution might be highly localized (e.g., in-area monitoring, like Target-Oriented WiFi Sensing for Respiratory Healthcare: from Indiscriminate Perception to In-Area Sensing), resulting in improving the recognition accuracy.

### 3.2 The wireless datasets

#### 3.2.1 ARWF wireless dataset

To evaluate the new personalized federated learning (PFL) algorithm, this study conducts simulations and experiments using two wireless datasets, with the target task being human activity recognition for wireless sensing in the future. The first wireless dataset is ARWF (Wang et al., 2019), which comprises 1,116 training samples and 278 test samples. Each sample features a spatial–temporal dimension of 52 × 192. In this dimension, 52 represents the number of subcarriers (a key parameter in wireless communication), and 192 corresponds to the number of time sampling points. For labeling, each sample is assigned 2 types of initial labels: 6 categories of human behavior and 16 categories of positions.

#### 3.2.2 Widar 3.0 dataset

The second wireless dataset, Widar3.0 (Zhang et al., 2021), is a typical wireless dataset that leverages Channel State Information (CSI) for sensing. Each sample in this dataset has a 3-dimensional structure of 2 × 1,000 × 90: specifically, 2 denotes the number of receiving antennas, 1,000 represents the number of time steps, and 90 corresponds to the number of subcarriers-all of which align with the inherent characteristics of CSI data. The dataset contains 6 gesture labels and is divided into 18 clients according to position to simulate the distributed nature of federated learning.

## 4 Results and discussion

In this section, we primarily evaluate the recognition accuracy of the proposed new clustered FL algorithm based on KL divergence (KLCFL) and its sensitivity to key factors based on wireless datasets.

### 4.1 Analysis of influencing factors of the new algorithm

The experiments primarily evaluate the effectiveness of the clustering and the effects of three key parameters on model training: *B* (the batch size of training data), *E* (local training rounds), and hyperparameter (clustering ratio) *cr*.

#### 4.1.1 Impact of batch size *B*

**Figure 1:**
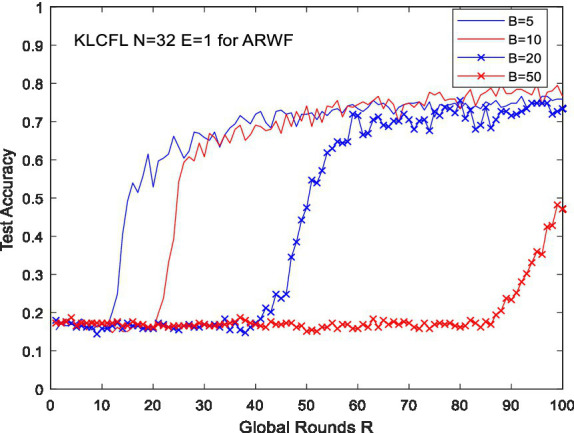
The effects of *B* on test accuracy based on ARWF.

For the ARWF dataset, under the conditions of a fixed number of clients *N* = 32, the number of global rounds *R* = 100, and *E* = 1, the test results are presented in Figure 1.

When *B* = 5, 10 and 20, the final test (recognition) accuracy of the KLCFL algorithm remains largely stable between 70 and 80% and exhibits relatively small fluctuations, as shown in Figure 1. When *B* = 5, the test accuracy of KLCFL increases rapidly at *R* = 10 or more, fluctuates slightly, and finally stabilizes at 80% at *R* = 100, as illustrated by the blue solid line in Figure 1. When *B* = 10, the test accuracy of KLCFL increases rapidly at *R* = 20 or more, fluctuates slightly, and finally stabilizes at 80% at *R* = 100, as illustrated by the red solid line. When *B* = 20, the test accuracy of KLCFL increases rapidly at *R* = 45 or more, fluctuates and finally stabilizes at 80% or so at *R* = 100, as shown by the blue star-solid line. However, when *B* = 50, the test accuracy of KLCFL begins increasing only at *R* = 90 and arrives at 55% or so at *R* = 100, as illustrated by the red star-solid line in Figure 1. In fact, *B* = 50 exceeds the total number of samples per client, which essentially amounts to using all samples in a single batch. This tends to make KLCFL overly fitted to the training set, ultimately leading to overfitting. Furthermore, a larger *B* typically results in smaller gradient variations, increasing the likelihood of KLCFL getting trapped in local optimal points. In contrast, a smaller *B* leads to larger gradient fluctuations, thereby reducing the likelihood of KLCFL getting stuck in local optimal points. It is also clear that the smaller the batch size *B*, the faster KLCFL converges, which in turn contributes to high recognition accuracy.

**Figure 2:**
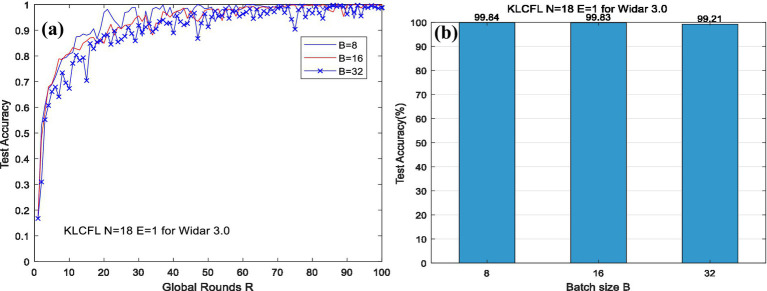
The effects of *B* on test accuracy based on Widar3.0 dataset (*a*/*b*).

For the Widar3.0 dataset, the number of clients *N* = 18. When *N* = 18 and *E* = 1, we investigated the impact of *B* (batch size) on KLCFL’s test (recognition) accuracy. Specifically, when *B* = 8, 16, and 32 (limited by the sample size of Widar 3.0 dataset), the corresponding test accuracies vary with global rounds *R*, as illustrated by Figure 2A. Although the trend affected by *B* is somewhat similar to that as mentioned earlier shown in Figure 1—the larger *B* is, the faster the convergence—the impact is insignificant and not obvious. Such discrepancies can be neglected, showing strong robustness. It is clear that as *R* increases, the recognition accuracy for different *B* values converges quickly and achieves excellent performance, exceeding 99%. At *R* = 100, the test accuracies are 0.9984, 0.9983, and 0.9921, respectively, as shown in Figure 2B. Although the test accuracy is relatively high—partly attributed to the high quality of the dataset—the influence pattern of *B* remains consistent: the smaller *B* value tends to relatively higher accuracy and faster convergence, and this kind of feature is affected by the quality of the dataset to a certain extent.

For these two datasets, this algorithm can converge quickly under different *B* values (*B* < 50), demonstrating strong robustness. For a given global round *R* (e.g., *R* = 100), the recognition accuracy of the algorithm KLCFL varies with different datasets, and the trend where the convergence speed tends to decrease as *B* increases also differs to varying degrees. Moreover, the convergence stability and amplitude fluctuation remain roughly consistent, with negligible differences within a certain range.

#### 4.1.2 Impact of local training round *E*

**Figure 3:**
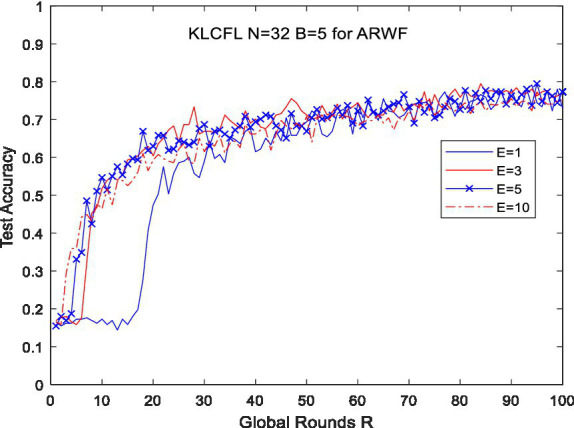
The effects of *E* on test accuracy based on ARWF.

For the ARWF dataset, let *N* = 32 and *B* = 5, we investigate the impact of *E* on KLCFL test accuracy. The results are presented in Figure 3.

The tested results demonstrate that as *E* increases (e.g., *E* = 1, 3, 5, 10), the recognition/test accuracy of KLCFL exhibits increasingly rapidly convergence with global rounds *R* until the recognition accuracy stabilizes at 0.8 or so. Additionally, the magnitude of fluctuations decreases progressively, while the overall performance remains stable, as shown in Figure 3. Under three different values of *E*, the differences in convergence accuracy and fluctuation amplitude are relatively small. However, there is a significant variation in convergence speed—with faster convergence observed as *E* increases (e.g., *R* < 20). Overall, local training rounds *E* exert little impact on recognition accuracy, indicating a certain degree of robustness.

**Figure 4:**
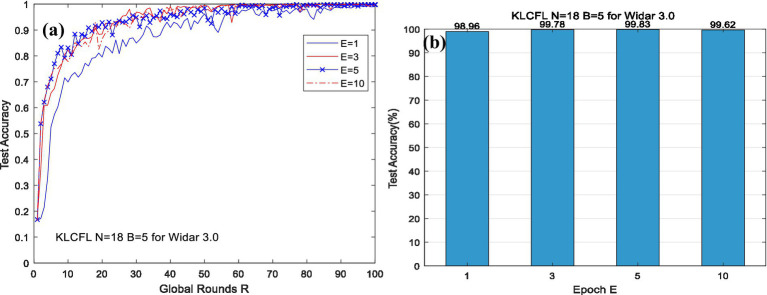
The effects of *E* on test accuracy based on Widar3.0 (*a*, *b*).

Similarly, for the Widar3.0 dataset, when *N* = 18 and *B* = 5, the KLCFL algorithm converges rapidly, as shown in Figure 4A. Additionally, it converges faster as *E* increases (e.g., *E* = 1, 3, 5, 10 at *R* < 20), although negligible differences still exist. Moreover, fluctuation magnitudes decrease progressively with *R* increasing and these differences can be negligible for *E* = 1, 3, 5.10. For a given global round *R* = 100, across all tested values of local training rounds *E* (e.g., *E* = 1, 3, 5, 10), the KLCFL algorithm achieves a high recognition accuracy exceeding 98%. This illustrates that the impact of local training rounds *E* on recognition accuracy is minimal and can be neglected, as shown in Figure 4B. In Figure 4B, when *R* = 100, the test accuracy of the KLCFL algorithm is 98.96, 99.78, 99.83 and 99.62% for *E* = 1, 3, 5.10, respectively. In terms of being affected by *E*, the KLCFL algorithm is less affected by it and exhibits greater robustness.

#### 4.1.3 Impact of clustering ratio *cr*

**Figure 5:**
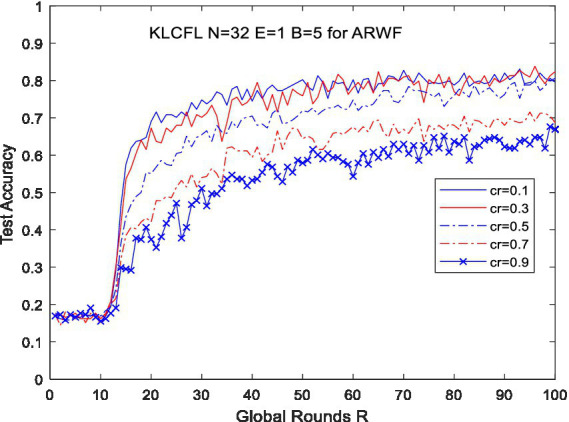
Curves of test accuracy variations of the KLCFL with different CR based on the ARWF dataset.

For the ARWF dataset, with *N* = 32, *E* = 1, and *B* = 5, we conducted simulation experiments by varying the clustering ratio (*cr*) of the KLCFL algorithm- defined as the ratio of the number of clustering operations to the total number of clients *N*. Specifically, *cr* × *N* denotes the number of clustering operation, with 9 clusters obtained when *cr* = 0.3. Figure 5 presents the curves illustrating test accuracy variations under different *cr* settings.

The new clustered federal learning algorithm KLCFL was tested with clustering ratio *cr* set to 0.1, 0.3, 0.5, 07, and 0.9 based on ARWF dataset. The test results indicate that KLCFL’s recognition/test accuracy increases as *cr* rises—particularly for *cr* <0.5—and maintains a relatively high level of approximately 80%, as shown in Figure 5. However, when *cr* = 0.7 and 0.9, the recognition accuracy begins to decline, dropping significantly to roughly 50%. This indicates that an excessively large *cr* exerts a substantial negative impact on model performance. A higher number of clusters means many clients with highly heterogeneous data may be grouped into the same cluster. This will inevitably lead to the same model being used to predict heterogeneous data, resulting in a decrease in recognition accuracy. It is clear that the performance of KLCFL degrades noticeably when the clustering ratio *cr* is excessively large. Therefore, the reasonable selection of the clustering ratio is of crucial importance, as it directly affects the algorithm’s recognition accuracy. From Figure 5, it is clear that the KLCFL algorithm can achieve satisfactory recognition accuracy when *cr* falls within the typical range of 0.01–0.5, demonstrating that the KLCFL algorithm exhibits moderate robustness in a certain degree.

**Figure 6:**
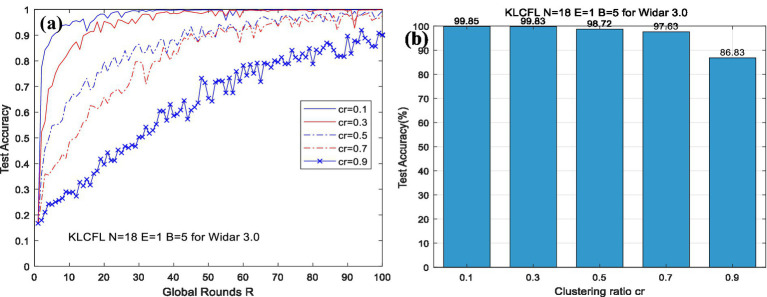
Curves of test accuracy variations of the KLCFL with different hyperparameters based on Widar3.0 (a, b).

For the Widar3.0 dataset with *N* = 18, as *cr* increases, the differences in the algorithm’s convergence speed become increasingly pronounced, as shown in the Figure 6A. The convergence speed of the algorithm becomes increasingly slow as the clustering ratio (*cr*) increases, and the accuracy also decreases—for example, the recognition accuracy of the KLCFL algorithm is 86.83% at *cr* = 0.9, and is more than 97% at *cr* <0.7, which is consistent with the previous simulation results. Therefore, the reasonable selection of the clustering ratio *cr* is of crucial importance, as it directly affects the algorithm’s recognition accuracy and convergence speed, as shown in Figure 6A. However, when *R* = 100, the algorithm achieves relatively high recognition accuracy (as high as 97%) for this dataset. Specifically, KLCFL achieves a recognition accuracy of 98% across all tested *cr* values (*cr* = 0.1, 0.3, and 0.5). Only when *cr* = 0.9 does the test accuracy decrease slightly, dropping to around 86%, as shown in Figure 6B.

Obviously, regardless of whether the dataset is of high or low quality, the clustering ratio largely affects the convergence speed and test accuracy. An excessively large clustering ratio will reduce the convergence speed and recognition accuracy. Therefore, the reasonable selection of the clustering ratio is crucial. For a dataset with unknown characteristics, it is generally feasible to refer to the test results of traversing multiple clustering ratios and select the optimal one by comprehensively considering the convergence speed and final recognition accuracy. Of course, for the two typical datasets selected in this study, choosing a clustering ratio below 0.5 is generally appropriate, which also ensures a certain degree of robustness.

### 4.2 Performance comparison between the new algorithm and several representative algorithms

To evaluate the performance of the new CFL algorithms (KLCFL) proposed in this study, we conduct a simulation-based performance comparison of KLCFL against several representative benchmark algorithms, including FedAvg (McMahan et al., 2017), FedRep (Collins et al., 2021), FedPer (Arivazhagan et al., 2019), PACFL (Vahidian et al., 2023; Aslam, 2023), and pFedMe (Dinh et al., 2020).

For the wireless dataset ARWF, we again observe that the KLCFL algorithm converges rapidly to notably high recognition accuracies: 83.05% (*N* = 16 defined by 16 positions) and 77.33% (*N* = 32, due to two clients at each position) at *R* = 100, as shown in Table 1. Among all the algorithms selected for comparison, the KLCFL algorithm proposed in this paper basically achieves the optimal/highest recognition accuracy and is worthy of application in practice. Table 1 presents the recognition accuracies of other six additional benchmark algorithms at *R* = 100 and *N* = 32, all of which are significantly lower than that of the KLCFL algorithm: (McMahan et al., 2017) a conventional FL algorithm, lacks sufficient consideration of personalization. FedPer (Arivazhagan et al., 2019) incorporates personalization, dividing the neural network model into base layers and personalized layers. The base layers are shared among all clients and updated by aggregation (e.g., by using FedAvg) on the server side. The personalized layers are trained only locally using the client’s own data, and do not participate in server-side aggregation. This architecture enables the model to both benefit from global collaboration and adapt to the specific data distribution of each client. The number of personalized layers (denoted as KP) is adjustable, which effectively addresses the data heterogeneity (Non-Independently and Identically Distributed-Non-IID) issue in federated learning. However, for FedPer (Arivazhagan et al., 2019) the precise partitioning of the “base layer and personalized layer” remains an open research question: the fixed division lacks theoretical basis and cannot be adjusted according to the training process or data characteristics. Moreover, the optimal KP value varies across different datasets and model architectures. To improve the recognition accuracy of FedPer, the basis for division and adaptive dynamic adjustment of KP should be performed according to changes in the training process and data characteristics. The algorithm pFedMe (Liang et al., 2022) encounters difficulties in selecting appropriate hyperparameters to quantify the degree of personalization. FedFomo (Zhang et al., 2020) biases personalized weights toward models with smaller losses. FedRep (Collins et al., 2021) fully addresses personalization (by designating the classification head as the personalized component) and effectively mitigates data heterogeneity in a certain degree. Its recognition accuracy is the closest to that of the KLCFL algorithm. The PACFL algorithm (Wang et al., 2019) primarily identifies distributional similarity by analyzing the principal angles between client data subspaces, which is called as cosine similarity.

**Table 1:** Test accuracy of KLCFL and several algorithms based on ARWF dataset (*R* = 100).

Algorithm	*N* = 16 clients	*N* = 32 clients
FedAvg	0.4590	0.5534
FedRep	0.8461	0.7926
FedPer	0.7978	0.7626
FedFomo	0.3849	0.4245
PACFL	0.6782	0.7334
pFedMe	0.6822	0.6547
KLCFL	0.8305	0.7953

After PCA vectors are normalized (e.g., low-dimensional representations), the Kullback–Leibler (KL) divergence can directly capture the “degree of information difference” between distributions. Cosine similarity (CS) only focuses on the consistency of vector directions, ignoring the difference in distribution intensity. In addition, in relation to the KL divergence, Euclidean distance (ED) merely measures the geometric spatial distance of vectors, failing to account for the probabilistic characteristics of distributions. Neither of CS and ED can address the core of the Non-IID problem: distribution deviation.

Additionally, KL divergence also has the advantage of effectively handling heterogeneous data. KL divergence is sensitive to the asymmetry and tail characteristics of distributions, effectively distinguishing between “distribution shift” and “pure numerical differences” represented by PCA vectors. Cosine similarity is insensitive to scale and ignores the distributional significance of the magnitudes of principal components in PCA vectors (e.g., the principal component magnitudes of a customer’s model concentrated in a few dimensions may correspond to specific distribution patterns). Euclidean distance is significantly affected by feature scales; even after PCA standardization, it may misclassify non-distributional numerical differences as heterogeneity. Moreover, the quantitative result of KL divergence directly reflects the “difficulty of distribution alignment,” enabling more accurate screening of customers with shareable model parameters after clustering. Cosine similarity may group customers “with consistent directions but significant differences in distribution shapes” into one cluster, while Euclidean distance may misclassify customers “that are geometrically close but distributional heterogeneous.”

**Figure 7:**
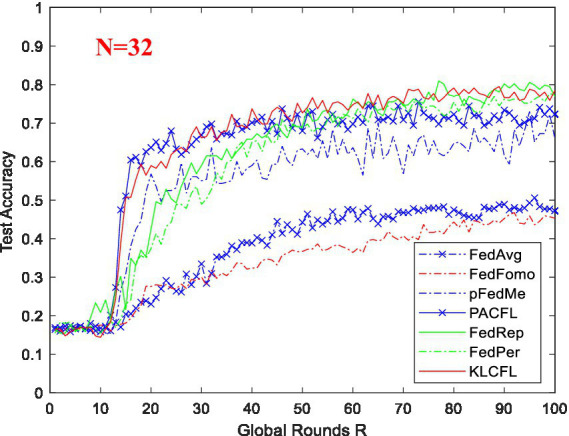
Test (recognition) accuracy of KLCFL and several representative algorithms based on ARWF dataset *N* = 32.

Figure 7 compares the convergence processes and recognition accuracies of the new algorithm KLCFL with several representative algorithms. It is evident that KLCFL basically outperforms those alternatives in four key aspects: recognition accuracy, convergence speed, stability, and amplitude fluctuation. In Figure 7, although the convergence speed of the KLCFL algorithm ranks the second (not the fastest) and its recognition accuracy is not the highest, its convergence process shows that the algorithm has a relatively fast convergence speed, a smaller jitter amplitude, and a relatively high final recognition accuracy, which is also relatively consistent with the ideal recognition accuracy. However, most other algorithms (such as FedAvg, FedFomo, and pFedMe) converge slowly and achieve much lower final recognition accuracy.

**Figure 8:**
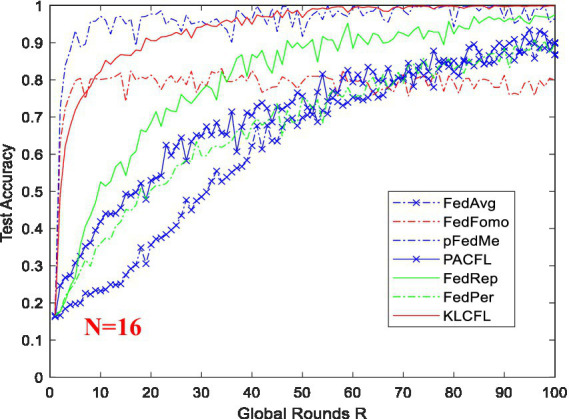
Test (recognition) accuracy of KLCFL and several representative algorithms based on Widar3.0 dataset.

Furthermore, to further verify the performance of the new algorithm, we again conducted tests using the Widar3.0 dataset. The test results demonstrate that by adjusting parameter *cr* (*cr* = 0.1), the KLCFL algorithm can effectively address data heterogeneity and achieve high recognition accuracy, as shown in Figure 8. Figure 8 presents a comparison of the recognition accuracies between the KLCFL algorithm and several other representative algorithms. It is clear that the recognition accuracy and stability of the KLCFL algorithm are significantly higher than that of the other several algorithms.

In Figure 8, the pFedMe algorithm converges relatively fast and achieves high recognition accuracy (0.965), but its amplitude fluctuation is somewhat significant—it still fluctuates when *R* reaches 100. The KL-based algorithm ranks the third fastest in convergence, with the smallest amplitude fluctuation, and stably converges to the final ideal recognition accuracy (0.998). FedFomo converges relatively fast, but its recognition accuracy is unsatisfactory (around 80%). FedRep exhibits a moderate convergence speed and good stability, with an acceptable final recognition accuracy (0.975). As for the other three algorithms, by comparison, they converge slowly and yield low recognition accuracy (FedFomo: 0.897, Fedavg:0.867, Fedper0.799) at *R* = 100.

In addition, on ARWF dataset, PACFL converges the fastest with small amplitude fluctuation and achieves excellent final accuracy. However, on Widar 3.0 dataset, it converges slowly with somewhat significant fluctuation, and the final recognition accuracy is not satisfactory. Similarly, pFedMe exhibits a moderate convergence speed on ARWF dataset but with relatively large fluctuation, and its final recognition accuracy is unsatisfactory and relatively low at *R* = 100. That is to say, these two algorithms show considerable variations in recognition performance across different datasets, indicating poor robustness. In contrast, the KLCFL algorithm proposed in this paper demonstrates the most outstanding recognition performance and stability on both datasets, boasting strong robustness and the ability to stably and effectively handle heterogeneous data.

Of course, the recognition accuracy of other algorithms may be improved to some extent through parameter optimization, but the KLCFL algorithm in this paper already achieves a relatively ideal high recognition accuracy. Even if one or two other algorithms are optimized and their accuracy may exceed that of this KLCFL algorithm, the improvement will be limited. In particular, compared with PACFL, a clustering algorithm of the same type with optimized parameters, the proposed algorithm exhibits higher accuracy and better convergence performance, effectively addresses the data heterogeneity issue, and possesses a certain degree of robustness.

## 5 Conclusion

Wireless data from different regions typically exhibits high heterogeneity, with limited labeled data available (details are reported in a separate study). Traditional FL struggles to achieve efficient and fast distributed model training under such circumstances. How to develop efficient training and adaptation methods for distributed wireless sensing models has become a major challenge in the development of 6G integrated communication and sensing networks. To tackle the challenges brought by heterogeneous wireless data, this study proposes an improved Personalized Federated Learning (PFL) algorithm.

This KLCFL algorithm incorporates the Kullback–Leibler (KL) divergence distance between each pair of clients, thereby enabling flexible clustering of clients with similar characteristics and significantly improving the recognition accuracy of wireless sensing in a great degree. For the ARWF dataset and Widar 3.0 dataset, KLCFL can all converges rapidly with small amplitude fluctuation and achieves excellent final accuracy, ranking among the excellent clustering algorithms.

Firstly, this paper carried out the PCA of the two datasets (ARWF and Widar3.0) and obtain a principal component vector matrix. And then it finished calculating the Kullback–Leibler (KL) divergence distance for clustering. Secondly, these two sets of wireless datasets were used to study the impacts of variations in batch size (*B*), local training epochs (*E*) and clustering ratio *cr* on the recognition accuracy of KLCFL. For the ARWF dataset, at *B* = 5 or 10, *E* = 2 or <4, and *cr* = 0.3 or <0.5, the KLCFL algorithm can achieve optimal recognition performance (77% ~ 83%). With respect to the Widar 3.0 Dataset, since it features relatively low interference levels and high data quality, the three key influencing factors exert minimal impact on the KLCFL algorithm’s recognition accuracy—thus enabling the KLCFL algorithm to exhibit good robustness. Generally, when *B* < 30, *E* < 10, and *cr* < 0.5, the KLCFL algorithm achieves a recognition accuracy of 98% or higher. Finally, the recognition accuracy of the KLCFL algorithm was compared and analyzed with that of several reported algorithms. For the ARWF Wireless Dataset, the recognition accuracy of the KLCFL algorithm reaches 83.05% (*N* = 16) and 79.53% (*N* = 32) when the global round *R* = 100, which is higher than those of other algorithms, as shown in Figure 7, Table 1. Moreover, KLCFL converges faster than other algorithms and exhibits smaller fluctuations in amplitude. For the Widar3.0 wireless dataset, the recognition accuracy of the KLCFL algorithm is 99%, outperforming several reported algorithms, as shown in Figure 8.

From Figures 7, 8, this KLCFL algorithm not only demonstrates high recognition accuracy and fast convergence, but also exhibits small fluctuations and high stability.

Based on these datasets KLCFL not only achieves higher recognition accuracy than the current reported algorithms, but also exhibits a faster convergence speed, smaller fluctuation amplitude, and greater stability during the convergence process.

It is clear that the proposed KLCFL is an excellent PFL algorithm that can effectively address the heterogeneity of wireless data and achieve high recognition accuracy.

Firstly, this KLCFL algorithm can quantify differences among heterogeneous distributions and provide theoretic support for the rationality of clustering: In federated scenarios, data from individual clients often exhibits Non-IID (Non-Independently and Identically Distributed) characteristics. KL divergence can accurately measure the asymmetric differences in data distributions across various clients, provide a quantitative basis for cross-client data clustering, overcome the limitations of traditional similarity metrics (e.g., Euclidean distance) regarding data distribution assumptions, and theoretically validate the feasibility of heterogeneous data clustering.

Secondly, the KLCFL algorithm can guide the direction of collaborative optimization for heterogeneous data: Based on clustering results derived from KL divergence, the distribution patterns of data heterogeneity in federated systems (such as the degree of local data deviation, variations in category distribution, and so on) can be revealed. This provides theoretical guidance for data partitioning and the collaborative updating of model parameters in federated training.

Finally, the KLCFL algorithm can improve the theoretical framework of federated clustering: Most existing federated clustering methods rely on the assumption of independent and identical data distribution. By integrating distribution difference measurement into the federated clustering framework, KL divergence clustering fills the theoretical gap in federated clustering for heterogeneous data and offers a referable theoretical paradigm for the design of clustering strategies in subsequent heterogeneous federated learning.

However, the Kullback–Leibler (KL) divergence is inherently asymmetric, which gives rise to unstable optimization trajectories during model training. Additionally, when two discrete distributions are non-overlapping or contain zero-probability entries, the KL divergence fails to effectively quantify the magnitude of their discrepancy—often resulting in undefined or infinite values. Furthermore, its robustness against noise, interference, and distribution shifts remains insufficient, posing significant challenges in real-world scenarios characterized by inherent perturbations.

To address these limitations, potential future research directions are outlined as follows: First, developing symmetric variants of the KL divergence (e.g., symmetric KL divergence or extensions inspired by the Jensen-Shannon divergence) to mitigate optimization instability induced by asymmetry. Second, adopting a minimal value substitution strategy (e.g., replacing zero entries with an infinitesimally small positive value) to avoid undefined results when zero-probability events occur. Third, when WiFi data exhibits distribution shifts due to dynamic environmental changes (e.g., personnel movement, signal occlusion), the KL divergence struggles to distinguish between “true class differences” and “distribution differences caused by scene interference.” This limitation is particularly prominent in dynamic scenarios and can be addressed by integrating improved KL measurement methods with scenario features and data characteristics, which may serve as a viable solution. Fourth, future research could focus on enhancing resilience against noise and interference by targeting physical attack scenarios—such as defending against physical layer attacks (PhyFinAtt) and keystroke sniffing (KeystrokeSniffer) (Liu et al., 2025; Chai et al., 2025). PhyFinAtt is an undetectable attack framework specifically designed to undermine PHY layer fingerprint-based WiFi authentication. KeystrokeSniffer demonstrates how an off-the-shelf smartphone can eavesdrop on keyboard input from anywhere. By applying PCA/KL to stabilize CSI features, KLCFL may make PHY fingerprints more resistant to environmental manipulation. By Clustering techniques may identify when an environment is being perturbed to attack PHY fingerprints, implementing online PCA updates would allow the system to continuously adapt to changing environments. For mitigation of Keystroke Sniffing, PCA/KL could transform WiFi signals in a way that obscures keystroke-related patterns. Clustering techniques could distinguish between harmless environmental variations and suspicious keystroke-related signal patterns, etc.

The proposed KLCFL and its PCA/KL approach reveal a promising direction for addressing the challenges of heterogeneous WiFi sensing data in security applications. By systematically reducing noise, stabilizing features, and clustering similar patterns, KLCFL could significantly enhance defenses against both PhyFinAtt and keystroke sniffing attacks.

## Data Availability

The original contributions presented in the study are included in the article/supplementary material, further inquiries can be directed to the corresponding author.
